# Pediatric Advance Care Planning for a Patient with a Severe Motor and Intellectual Disorder through Cooperation between an Acute Care Hospital and a Residential Facility

**DOI:** 10.1089/pmr.2021.0050

**Published:** 2022-06-07

**Authors:** Hiroshi Wada, Masahisa Funato, Kiyoshi Takemoto, Yoshitaka Iijima, Atsuko Kashiwagi, Natsuko Shiomi, Shigetoyo Kogaki, Tomoko Maruyama

**Affiliations:** ^1^ Department of Pediatrics of Osaka Developmental Rehabilitation Center, Osaka, Japan.; ^2^ Department of Pediatrics of Osaka General Medical Center, Osaka, Japan.

**Keywords:** advance care planning, medical complexities, multifacility collaboration, severe motor and intellectual disabilities

## Abstract

**Background:**

With the rapid progress of medical technology, the number of children with medical complexities who require advanced medical care, including mechanical ventilators, has been increasing steadily in Japan. Accordingly, the issue of how to provide holistic care and support for the entire life of the children with severe motor and intellectual disabilities (SMID) who live at home has become a new challenge.

**Case Presentation:**

We present the case of a three-year-old boy with SMID due to HHV-6B-induced hemorrhagic shock encephalopathy who was cared for at home by the home visit medical team of Osaka Developmental Rehabilitation Center (ODRC; residential facilities with the department of home medical treatment and care). He developed septic shock triggered by an urinary tract infection and was admitted to Osaka General Medical Center (OGMC; acute care facility not directly affiliated with ODRC), where he deteriorated to a terminal stage. After discussing advance care planning (ACP) with his parents, along with the medical team, an ACP document with parental wishes was created through collaboration between the two facilities. The document was approved by the Ethics Committee at OGMC and the parents signed the document. Special end-of-life care planning was given by nurses at OGMC based on the best interests of the patient and the family. The patient passed away peacefully surrounded by his family in a private room of OGMC according to the ACP, despite special limitations caused by the coronavirus disease 2019 (COVID-19) pandemic.

**Conclusions:**

ACP provides a good opportunity to think about the best total care for a child with SMID, for whom it is too difficult to express his or her wishes, together with the parents, who are the legal representatives. The collaboration between two institutions with different roles brought out the best of each, and the resulting ACP was beneficial to the patient and their family.

## Introduction

Unlike with cancer and dementia in adults, terminal care for children often lasts for a long time.^1^ In particular, children with severe motor and intellectual disabilities (SMID) require multidisciplinary support not only at the end of life, but also in their daily life while supporting of their development, education, life transitions, and independence. With the rapid progress of medical technology, the number of children who require advanced medical care, such as children with chronic complex conditions has been increasing steadily in Japan. Accordingly, the question of how to provide holistic care and support for the entire life of the child through multidisciplinary work in cases when the child is discharged from the neonatal intensive care unit (NICU) and starts living at home has become a new issue.

In Japan, advance care planning (ACP) is currently not sufficiently addressed. The Ministry of Health, Labor, and Welfare (MHLW) began referring to ACP in 2018 as a “Life Conference” and has been working to promote its use. This is especially the case for elderly people or patients with malignant diseases. There have been some successful responses in adult patients with severe illnesses for whom Do Not Attempt Resuscitation is indicated, but efforts have not advanced further or become widespread, especially in children with malignant diseases or with SMID. In addition, guidelines for discussions about children with serious illnesses are often checklist based, and individualized care plans are rarely discussed in Japan.^2^ In short, there is much work to be done.

Home care medicine for children with medical complexity (MC) is now more commonly supported by district or visiting physicians, nurses, rehabilitation therapists, care providers, and so on. However, there is still too little information and discussion on how to achieve the best interests of the patient and families. In this context, we have continued providing end-of-life care with ACP for these children with MC in Japan.

Our Osaka Developmental Rehabilitation Center (ODRC) is a comprehensive facility that provides medical and welfare support such as service coordination by a medical social worker, nursery kindergarten, and individual nursing by caregivers for children with MC. We build a network for supporting children with disabilities and provide appropriate medical support through medical treatment and nursing at home. In this context, we have continued our activities as one of the few facilities that offers pediatric home care in Japan. However, in the event of a sudden deterioration in the condition of the patients at home, referral for hospitalization at a collaborating acute care or emergency hospital is required, as ODRC is a residential chronic care facility. In some cases, we could not discuss with the families the best way to proceed with peaceful end-of-life care because they want the maximum amount of invasive treatment.

In this article, we present the case of a patient whose ACP was created by collaborating between two facilities. After the staff of ODRC supported the patient and his family at home, he was admitted to a tertiary facility due to a sudden deterioration in his condition. Afterward, an ACP document was created in accordance with the family's wishes, leading to comfort-focused end-of-life care.

We obtained approval for the ACP proposal and this article from the Ethics Committee of our Center (Rin 20-5). We also obtained consent and approval from the family for this article.

## Case Presentation

We present the case of a 3 year 10-month-old boy. At the age of 1 year and 11 months, he had a high fever of over 40°C and tonic convulsions. He was brought to the emergency room of a tertiary emergency hospital in another prefecture. After close examination, he was diagnosed with hemorrhagic shock encephalopathy syndrome caused by HHV-6B. His general condition stabilized after two weeks of inpatient treatment, but his spontaneous breathing was poor and the function of his pituitary gland was severely impaired. This meant he required 24-hour ventilation and tube feeding. His Glasgow Coma Scale was 3 and he could not respond to external stimulation. The parents, however, strongly desired home care and they wished for him to receive as aggressive treatment as possible, including resuscitation, if his condition deteriorated. The family requested aggressive care because there were many things remaining that they wanted to do with their son.

After intensive care at the hospital, he was transferred to ODRC for support and preparation of home care. After the parents moved to Osaka, he was discharged from ODRC after receiving three months of care from our multidisciplinary support program, and transitioned to our home care program. Then we, the home medical support team of ODRC, began home medical treatment, nursing, rehabilitation, and home care. At that time, the wishes of the parents had not changed; they wished him to receive as aggressive treatment as possible, including resuscitation.

The patient and the family were supported by the home support team, as shown in Table 1. Intensive treatments of ventilation therapy, tube feeding, and regular medication were continued at home, and he began to use the short-stay service at our ODRC as well as the day care service. He was stable and did not require additional oxygen administration, antimicrobial medications, or hospitalization. However, one day, almost one year after his transition to home care, his condition rapidly deteriorated, and his heart rate increased, oxygen saturation decreased, and urine output decreased. Peripheral circulatory failure was also observed, and he was diagnosed with the condition of preshock. We requested hospitalization at Osaka General Medical Center (OGMC), an acute hospital, and he was transported there for the emergency. A urinary tract infection led to septic shock. He was treated in the intensive care unit. The treatment was successful and he was discharged one month later.

**Table 1.** Organization of Home Support Team

(1) Visiting nursing: two Visiting Nursing Stations A and B, visiting four days a week
(2) HMT: Department of HMT of ODRC, visiting twice a month regularly and having emergency visit with 24 hours response
(3) Emergency treatment for admission: OGMC
(4) Visiting rehabilitation: two Visiting Nursing Stations, visiting three days a week
(5) Visiting care: one Visiting Care Station, visiting two days a week
(6) Short stay: ODRC
(7) Developmental support center for children
(8) Counselling and support center

HMT, home medical treatment; ODRC, Osaka Developmental Rehabilitation Center; OGMC, Osaka General Medical Center.

**FIG. 1.**
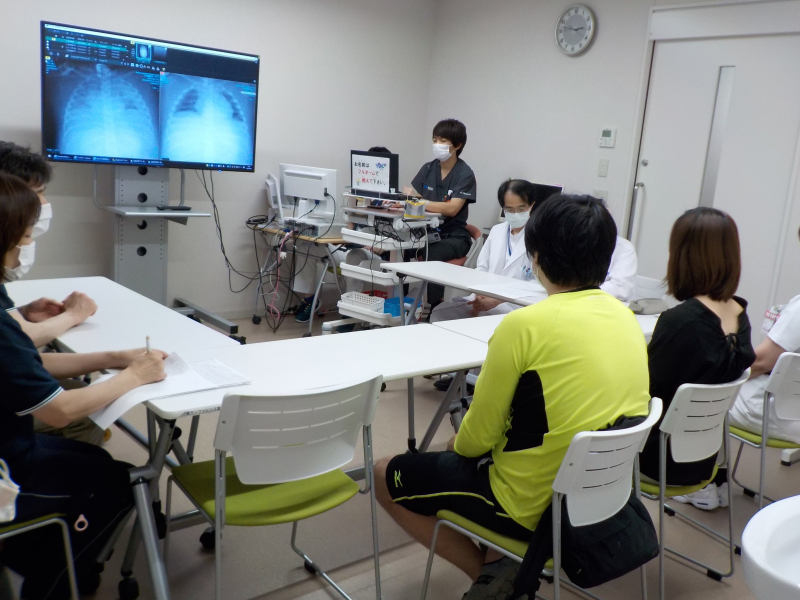
Photo of the conference with the staff of two facilities and the parents of the patient.

However, the patient was readmitted to OGMC again five days later with a worsened general condition, caused by a respiratory infection. Oxygen administration had to be increased, respiratory settings had to be raised, and tube feedings could not proceed due to gastrointestinal dysfunction, so the intravenous hyperalimentation, which had been started at the beginning of his admission, could not be discontinued. After consultation with the OGMC physicians, we suggested that we discuss next steps and what would be in the child's best interest if his condition worsened and recovery became difficult. The parents, who had been hoping for a full resuscitation in the event of deterioration, agreed to this proposal after discussion with us; they accepted the full invasive intervention was not always good to the best interests of the patient. A basic document was exchanged between the staff of ODRC and OGMC. After the initial ACP was drafted, physicians and nurses of ODRC went to the OGMC and had several further discussions with the family (Fig. 1). The draft was amended, incorporating the wishes of the parents (Table 2), and it was approved by the OGMC Ethics Committee and signed by the parents for completion.

**Table 2.** The Drafted Advance Care Planning Incorporating the Wishes of the Parents

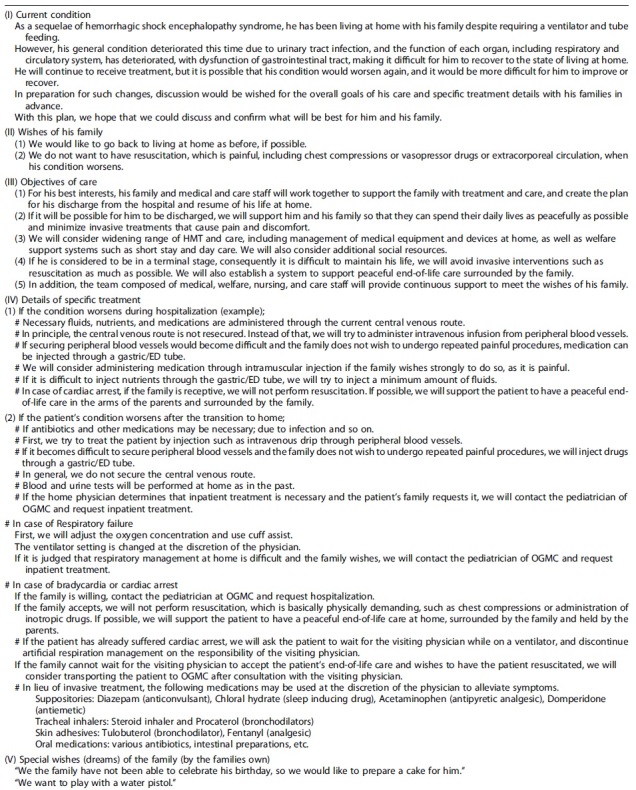 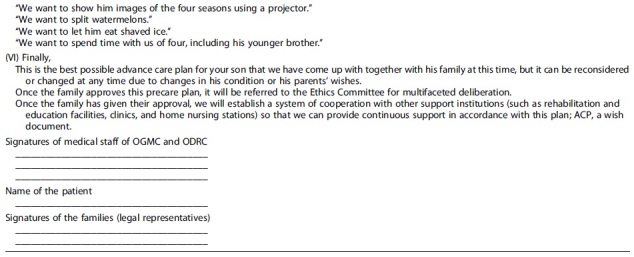

ACP, advance care planning.

During his hospitalization at OGMC, photos were taken of the patient, he was bathed for comfort, and his nurses and childcare staff made artworks with his handprints and footprints for the family. Although there were restrictions on visits due to the coronavirus disease 2019 (COVID-19) pandemic, the patient could be moved from the high care unit to a private room, and we were able to fulfill the family's wish to have visits from his sibling and relatives, as well as celebrate his birthday, which had not been possible due to the exacerbation of his physical condition. Although the parents strongly wanted to take the patient home, they could not because the ventilator was set too high to be changed to the usual one at home. The child passed away peacefully shortly thereafter, surrounded by his family. His family received grief support from our team.

When we, the ODRC home visit team, visited the bereaved family three months and one year after his death, his family expressed their gratitude again and said, “We did the best we could, and we have no regrets. The days of taking care of him at home were truly a blessing. We were really happy to be able to see our child, at home” (Table 3).

**Table 3.** The Words of the Family When We Visited the Home One Year after His Death

(1) The days we were able to take care of him at home were truly a blessing.
(2) There were so many things we wanted to do, and there was no end to what we could have done, but we were able to do the best we could, and we have no regrets now.
(3) During the last week, his father took a day off from work and we were able to spend some time together as a family. I am very grateful that the hospital was kind enough to allow our family to visit him under the COVID-19 epidemic. Although his death was painful, there are no regrets in his end for us families.
(4) It was really hard right after he passed away, but the whole family has been able to stay healthy throughout the year, and we have been able to spend our time together more peacefully than we expected. His little brother has been a big help to me. His presence has helped me to stay positive.

COVID-19, coronavirus disease 2019.

## Discussion

In Japan, the MHLW developed the “Guidelines for Decision-Making Process for Terminal Care” in 2007 and published the “Guidelines for Decision-Making Process for Medical Care in the Final Stage of Life” in 2015. These guidelines have been applied to end-of-life care for adults, but there are still few cases in which these guidelines are applied for children, and even less so in children with SMID.

According to a questionnaire survey of facilities for severely disabled children in the Kinki region (around Osaka prefecture), and residential facilities participating in a physician exchange meeting conducted in 2017, 18 facilities reported a total of 69 cases of end-of-life care experiences in the past five years.^3^ Of these, 15 were under the age of 18, and the largest number of cases were those in which the decision making for those who were unable to express their wishes was done through collaboration between the family and the medical/care team. In cases where the patient was dying, special care was given to the families, including limiting invasive treatment, providing a private room, having the family participate in the care, and introducing palliative care. The most common way of documenting the content of discussions with families was to write it in the patient's medical chart, and only two facilities created an ACP document. It was found that residential facilities for children with disabilities are advancing end-of-life care gradually by discussing the best interests of the patient with the family, who are the legal representatives, and the medical/care team.

Recently, the application of ACP has expanded worldwide to critically ill children whose prognoses are limited and who are unable to express their own wishes.^4–7^ In the National Institute for Health and Care Excellence (NICE) guideline, advance care planning needs to include children and young people with a life-limiting condition, and consideration should be made for the child/young person and family's beliefs and values.^8^ In the United Kingdom, palliative care is informed by the guidelines so that consultation is done through a sequential analytical process.^9^ On the other hand, the MHLW issued two Guidelines for Decision-Making Process in Japan as mentioned before, and the latter was revised in 2018 so that ACP is now especially recommended for the elderly and those in a weak condition.

In 2014, our center ODRC reported the case of a child with a very serious illness whose end-of-life care was provided according to the ACP for the first time in facilities for patients with SMID in Japan.^10^ Since then, we have been attempting to discuss and make collaborative decisions about how to respond to sudden changes in the patient's condition, which may be life-threatening, by focusing on the patient's dignity and best interests. The content of the care plan is discussed in collaboration with a multidisciplinary team and their feedback is incorporated as much as possible when the ACP draft is prepared. Based on this draft, the family discusses and freely modifies the plan, signs it, and submits it as a wish document to the Ethics Committee for multifaceted review and approval. If approved, we provide palliative care along with proper medical care and educational support for the family, and the staff.

During our initial support program for transition to home care, the family in this case wanted maximum aggressive treatment in case of the patient's deterioration. After discharge, his condition stabilized with the support of visiting physicians, nurses, and other staff, and the policy was not changed. However, there was a sudden change in his condition after one year so that he needed intensive care again at OGMC. A new policy was required in the process of explaining the condition of the child to the family. So, we the physicians of ODRC visiting team, went to OGMC to discuss the best interests of the child and to create an ACP plan together.

After obtaining the family's consent, we sent a draft of the ACP to the OGMC physician, and we, the ODRC staff, visited OGMC several times to review and refine the document with the family. The ODRC physician visited OGMC several more times and worked with the family on the document, which was approved by the OGMC Ethics Committee and signed by the family. Even under the severe situation of coping with the COVID-19 pandemic from the end of 2019, the nurses and nursery staff of OGMC were able to lead the care of the child under safe management, and the child's family and relatives were able to spend a meaningful time in a relaxed atmosphere with the patient, while he was cared for in a loving manner. Although the parents' strong wish to return to their home with the patient was not medically feasible, the family expressed their gratitude to the two facilities after his death.

In Japan, acute hospitals usually focus on intensive care and do not consider palliative care, especially in the case of acute exacerbations of severe illnesses such as SMID. We believe that it is significant that the ODRC has been able to share with acute hospitals our approach to the best interests of SMID on a daily and lifelong basis, and to jointly produce the ACP. The ACP is a wish document, and its purpose for children, especially those with SMID, is to support them and their families by uniting the thoughts of those involved based on the document.

## Conclusion

The role of medical treatment and education is to provide individualized support for the best interests of the child while respecting the child's and family's human rights and dignity through the medical and care team. End-of-life support with palliative care and the ACP process provides an excellent opportunity to the best holistic care for the child.

## Abbreviations Used

ACP: advance care planning
COVID-19: coronavirus disease 2019
HMT: home medical treatment
MC: medical complexity
MHLW: Ministry of Health, Labor, and Welfare
NICE: National Institute for Health and Care Excellence
ODRC: Osaka Developmental Rehabilitation Center
OGMC: Osaka General Medical Center
SMID: severe motor and intellectual disabilities
